# Calendar

**DOI:** 10.1155/S146392467900033X

**Published:** 1979


devices, together with other equipment needed to program them.

The booklet is available from Microsystem Services, Duke Street, High Wycombe, Bucks., U.K.

### Stepping motors

Unimatic Engineers Ltd., now have available the new catalogue from Sigma Instruments Inc., detailing their complete range of stepping and synchronous motors. The catalogue gives full technical information on the motors and also provides applications advice, performance curves and application considerations. Information on Sigma's range of stepping motor drives and controllers is also included. It provides formulae for the calculation of torque and inertia figures coupled with metric/imperial conversion tables for both torque and rotary inertia.

The catalogue is available, free of charge, from Unimatic Engineers Ltd., Granville Road Works, 122 Granville Road, Cricklewood, London NW2, U.K.

### Pollution monitoring equipment

The latest 16-page catalogue from Techmation describes the range of pollution monitoring instrumentation available from a variety of manufacturers: Meloy, Dohrmann, Axel Johnson, Ionics, Turner Designs, Manning and Spectrametrics.

Instruments are described to monitor sulphur gases, oxides of nitrogen, phosphorus, ozone, hydrocarbons and purgeable halides. Total organic sulphur, chloride and nitrogen monitors and calibration sources are also included.

Water pollution monitoring equipment covers a wide range: total organic carbon, total carbon, total oxygen demand and organic contamination monitors; for physical measurements, fluorometers, nephelometers, flow and level recorders and sampling devices. Elemental analysis by plasma emission spectroscopy completes the range.

Free copies of the catalogue are available from Techmation Ltd., 58 Edgware Way, Edgware, Middx., U.K.

### User guide to GASP

The application of a computer program for the analysis of gamma-ray spectra (GASP) at Harwell is described at a level suitable for users with practical experience of spectrometry, but limited knowledge of computer methods in a document published recently by HMSO, price £1.50. It is designed to help users of GASP, written in Fortran language, to be aware of the facilities available.

'A desktop computer system to control Auger Emission Spectroscopy' is the title of another document, price £1.00, published by HMSO to describe how a simple modular interface has been constructed to allow a Hewlett-Packard 9845 desktop computer to control the data acquisition from an Auger Electron Spectrometer.

Both documents are available from HMSO, PO Box 569, London SE1 9NH, U.K.

### Tool and production aid catalogue

The first edition of the Toolrange catalogue, aimed specifically at the electrical industries and containing a comprehensive range of tools, toolkits and production aids, is now available. A mail order service is provided and it offers users access to many selected items from over 60 British and international manufacturers. The catalogue is divided into twelve major product groups from soldering and desoldering through to welding and brazing.

Further details and copies of the catalogue can be obtained from Toolrange Ltd., Upton Road, Reading, Berks. RG3 4JA, U.K.

### Liquid chromatography catalogue

A 44-page catalogue is available from Laboratory Data Control providing full details of their entire product range. The range provides almost every item of equipment required for high performance liquid chromatography from tubing to a complete chromatograph. The catalogue details the main equipment range with operating specifications and ordering information. It is divided into four main sections; complete chromatographs, complete modular systems, individual modules, valves, columns and accessories. A final section contains applications data and advice.

The catalogue is available from European Office, 52 High Street, Stone, Staffs. ST15 8AR, U.K.

### Calendar

### 1979

**The Applications of Microprocessors: A review lecture by Professor J. H. Westcott**
February 8, London, U.K.
*The Executive Secretary, The Royal Society, 6 Carlton House Terrace, London SW1Y 5AG, U.K.*

**Clinical Laboratory Testing in Europe**
February 12-13, London, U.K.
*Robert S. First, Inc., 405 Lexington Ave., New York, 10017, U.S.A.*

**Use of the Microprocessor as a Component in Measurement and Control Equipment**
February 13-15, Stevenage, Herts., U.K.
*Carole Meads, Sira Institute, South Hill, Chislehurst, Kent, U.K.*

**30th Annual Conference on Analytical Chemistry and Applied Spectroscopy**
March 5-9, Cleveland, U.S.A.
*Mrs. L. Briggs, 437 Donald Road, Pittsburgh, Passadena 15235, U.S.A.*

**Process Analytical Instrumentation**
March 19-23, Coventry, U.K.
*Deputy Secretary, The Institute of Measurement and Control, 20 Peel Street, London W8, U.K.*

**International Conferences on Spectroscopy**
March 29-30, Miami, U.S.A.
*Vijay Mohan Bhatnagar, Alena Enterprises of Canada, P.O. Box 1779, Cornwall, Ontario, Canada K6H 5V7.*

**Kongress der Deutschen Gesellschaft fur Laboratoriumsmedizin**
March 29-April 3, Berlin, Germany.
*Wilhelm Syborg, Kongresshall Berlin, John-Foster-Dulles-Allee 10, 1000 Berlin 21, West Germany.*

**River Pollution Control**
April 9-11, Oxford, U.K.
*Water Research Centre, Stevenage Laboratory, Elder Way, Stevenage, Herts. SG1 1TH, U.K.*

**3rd International Symposium on Capillary Chromatography**
April 30-May 3, Hindelang/Allgau, W. Germany.
*R. E. Kaiser, P.O. Box 1308, D-6702 Bad Durkheim 1, Germany.*

**9th Annual Symposium on the Analytical Chemistry of Pollutants**
May 7-9, Georgia, U.S.A.
*Elaine McGarity, Environmental Protection Agency, Environmental Research Laboratory, College Station Road, Athens, Georgia 30605, U.S.A.*

**Current Developments in the Clinical Applications of HPLC, GC and MS**
May 30-June 1, Harrow, U.K.
*Symposium Secretariat, Division of Clinical Chemistry, Clinical Research Centre, Harrow, Middlesex, U.K.*

**3rd European Congress of Clinical Chemistry**
June 3-8, Brighton, U.K.
*Dr. F. Mitchell, CRC, Northwick Park, London, U.K.*

**3rd European Congress of Clinical Chemistry**
June 4-8, Brighton, U.K.
*Dr. P. J. N. Howarth, Department of Chemical Pathology, Guy's Hospital, Medical School, London SE1 9RT, U.K.*

**2nd World Chromatography Conference**
June 5-6, Lisbon, Portugal.
*V. M. Bhatnagar, P.O. Box 1779, Cornwall, Ontario K6H 5V7, Canada.*

**10th International Symposium on Chromatography and Electrophoresis**
June 19-20, Venice, Italy.
*Dr. Alberto Frigerio, Instituto di Riccerche Farmacologiche "Mario Negri," Via Eritrea 62, 20157 Milan.*

**8th International Conference on Atomic Spectroscopy**
July 1-6, Cambridge, U.K.
*Association of British Spectroscopists, P.O. Box 109, Cambridge CB1 2HY, U.K.*

**2nd Canadian World Chromatography Conference**
July 5-6, Toronto, Canada.
*V. M. Bhatnagar, P.O. Box 1779, Cornwall, Ontario K6H 5V7, Canada.*

**Summer School on Automatic Methods of Analysis**
July 9-13, Swansea, U.K.
*D. Porter, Laboratory of the Government Chemist, Cornwall House, Stamford Street, London SE1 9NQ, U.K.*

**Analysis '79: Automation in Industrial and Clinical Chemistry**
July 16-18, London, U.K.
*Scientific Symposia Ltd., 33-35 Bowling Green Lane, London EC1R 0DA, U.K.*

**3rd International Bioanalytical Forum**
September 4-7, Guildford, U.K.
*Dr. E. Reid, Wolfson Bioanalytical Centre, University of Surrey, Guildford, GU2 5XH, U.K.*

**Automation and Computerisation in the Medical Laboratory**
September 5-7, Stirling University, Scotland.
*G. W. Thomson, Secretary, IMLS Glasgow Branch, 67 Glen Ogilvie, St. Leonards, East Kilbride, Scotland.*

**International Conference on Flow Analysis**
September 11-13, Amsterdam, The Netherlands.
*Secretary FA-Amsterdam, Laboratory for Analytical Chemistry, University of Amsterdam, Nieuwe Achtergracht 166, 1018 WV Amsterdam, The Netherlands.*

**Communications in Microprocessor Industrial Instrumentation**
September 12-13, London, U.K.
*Mrs. P. Keiller, Sira Institute, South Hill, Chislehurst, Kent BR7 5EH, U.K.*

**Le Chromatographe Automatique Industriel en Lague: Analyseur Sophistique ou Capteur Industriel?**
October 3-5, Arles, France.
*Institute de Regulation et Automation Guy Berthier, Chemin des Moines, 13644, Arles, France.*

**Real-time Datahandling and Process Control**
October 23-25, Berlin, West Germany.
*Congress Organisation Company, John Foster Dulles Allee 10, D-1000 Berlin, West Germany.*

### 1980

**Automation at SAC 80**
July 20-26, Lancaster, U.K.
*The Secretary, Analytical Division, The Chemical Society, Burlington House, London W1Y 0BN, U.K.*

### 1981

**Euroanalysis IV**
August 23-28, Helsinki, Finland.
*Association of Finnish Chemical Societies, Executive Secretary, Pohj, Hesperiankatu 3B10, SF-00260 Helsinki 26, Finland.*

**Editor's Note:**
*Organisers of conferences, seminars etc. should send details for inclusion in this calendar as soon as the relevant information is available and not later than three months before the event.*

### Product News

### Laboratory instrumentation and equipment

ChemLab Instruments Ltd., will be exhibiting a selection of items from their wide range of laboratory instrumentation and equipment at Labex '79, 12-16 March 1979. Several new products are to be shown at this exhibition.

A new peristaltic pump with stand-by facility to reduce reagent consumption and a new design of analytical cartridges using AAII type chemistries have been added to the well-established range of equipment for continuous flow analysis. The new Triton 3 micro-computer for use as a general purpose laboratory data processor will also be on display. Amongst the increasing range of analytical programmes is a system for handling simultaneously the results from a number of different gas chromatographs and high performance liquid chromatography.

Other products that will be on display include Nichiryo dispet and micro-dispet dispensers, an improved range of ultrafiltration equipment, Brinkmann probe colorimeters, Wescor osmometers, the Prolabo HPLC system and the W.T.W. Combibox from Germany, which is for checking continuously pH, conductivity and temperature of water effluents.
*ChemLab Instruments Ltd., Hornminster House, 129 Upminster Road, Hornchurch, Essex RM11 3XJ, U.K.*

### Measurement of blood clotting

Blood clotting disorders can now be studied more specifically using a series of methodologies made available recently.

These methodologies use a synthetic chromogenic substrate enabling specific photometric measurement of coagulation chemistries to be done with ease. There are methods for evaluations of antithrombin III, herapin and antiplasmin.

Automation of these tests are specifically suited to the Vitatron PA800, a fully automated programmable clinical analyser. For smaller workloads the Vitatron Reaction Rate Photometer (RRP) is ideal. This instrument when used in conjunction with high precision dispensers and diluters for reagent addition enables accurate measurement of both high and low concentrations of blood clotting factors.
*MSE Scientific Instruments, Manor Royal, Crawley, Sussex RH10 2QQ, U.K.*

### Optical system

The InfraAlyzer 400 is the result of three years research and development and is a natural heir of the earlier InfraAlyzer models from Technicon Industrial Division. The system operates in 8 seconds automatically and incorporates the infra-vision screen which allows commodity recognition. It operates with up to 20 filters instead of the original 6, so allowing a wider range of products to be analysed making it ideal for research into new applications.

The optical system incorporates an integrated sphere detector which provides continuous reference correction and high signal to noise ratios. The essential benefits are accuracy, no drift and faster performance.
*Technicon Instruments Co. Ltd., Evans House, Hamilton Close, Basingstoke, Hants. RG21 2YE, U.K.*

